# Repair of osteochondral defects: efficacy of a tissue-engineered hybrid implant containing both human MSC and human iPSC-cartilaginous particles

**DOI:** 10.1038/s41536-023-00335-x

**Published:** 2023-10-19

**Authors:** Shinichi Nakagawa, Wataru Ando, Kazunori Shimomura, David A. Hart, Hiroto Hanai, George Jacob, Ryota Chijimatsu, Seido Yarimitu, Hiromichi Fujie, Seiji Okada, Noriyuki Tsumaki, Norimasa Nakamura

**Affiliations:** 1 Department of Orthopaedic Surgery, Osaka University Graduate School of Medicine, Suita, 565-0871 Japan; 2 Department of Orthopaedic Surgery, Kansai Rosai Hospital, Amagasaki, 660-8511 Japan; 3 McCaig Institute for Bone and Joint Health, Department of Surgery and Faculty of Kinesiology, University of Calgary, Calgary, AB T2N 4N1 Canada; 4 Department of Medical Data Science, Osaka University Graduate School of Medicine, Suita, 565-0871 Japan; 5 Department of Mechanical Systems Engineering, Faculty of Systems Design, Tokyo Metropolitan University, Hachioji, 192-0364 Japan; 6 Department of Clinical Application, Center for iPS Cell Research and Application, Kyoto University, Kyoto, 606-8507 Japan; 7 Department of Tissue Biochemistry, Graduate School of Medicine and Frontier Biosciences, Osaka University, Suita, 565-0871 Japan; 8 Institute for Medical Science in Sports, Osaka Health Science University, Osaka, 530-0043 Japan; 9 Center for Advanced Medical Engineering and Informatics, Osaka University, Suita, 565-0871 Japan

**Keywords:** Regenerative medicine, Tissue engineering, Mesenchymal stem cells

## Abstract

Both mesenchymal stromal cells (MSC) and induced pluripotent stem cells (iPSC) offer the potential for repair of damaged connective tissues. The use of hybrid implants containing both human MSC and iPSC was investigated to assess their combined potential to yield enhanced repair of osteochondral defects. Human iPSC-CP wrapped with tissue engineered constructs (TEC) containing human MSC attained secure defect filling with good integration to adjacent tissue in a rat osteochondral injury model. The presence of living MSC in the hybrid implants was required for effective biphasic osteochondral repair. Thus, the TEC component of such hybrid implants serves several critical functions including, adhesion to the defect site via the matrix and facilitation of the repair via live MSC, as well as enhanced angiogenesis and neovascularization. Based on these encouraging studies, such hybrid implants may offer an effective future intervention for repair of complex osteochondral defects.

## Introduction

Articular cartilage plays an important role in joint lubrication and function. However, articular cartilage is both avascular and aneural, as well as has a cellularity of low density^1^. These characteristics likely contribute to a compromised healing capacity. Chondral injury often extends to subchondral bone pathology^2,3^ and can progress to osteoarthritis which affects both the cartilage and the underlying bone. Therefore, an effective approach for the biphasic restoration of cartilage and subchondral bone is required for the successful repair of osteochondral lesions^4–6^ and the prevention of further progression to develop overt osteoarthritis. Many biological and cell-based approaches have been assessed to improve osteochondral repair, using several different cell sources^7–10^, and some have used constructs with cells + artificial bone to affect osteochondral repair in preclinical models such as rabbits^9^.

Induced pluripotent stem cells (iPSCs) can be prepared from mature somatic cells^11–13^ and implanted iPS cells could potentially mediate bone and cartilage repair. iPSC-derived cartilaginous particles (iPSC-CP), which have a hyaline-cartilaginous matrix with low tumorigenicity can be generated via in vitro differentiation^14^. Thus, iPSC-CPs have the potential to contribute to the repair osteochondral tissues^14^. Conversely, due to the nature of the hyaline-like matrix, iPSC-CPs themselves are not adhesive and may require reinforcement (e.g., fibrin glue) for fixation when implanted into a large osteochondral defect^10^.

We have previously developed a scaffold-free three-dimensional tissue-engineered construct (TEC) composed of synovial mesenchymal stromal cells (MSC) and extracellular matrices (ECM) synthesized by the cells and which are free from any artificial or extrinsic biological materials^15,16^. Such TEC have strong tissue adhesive properties and plasticity, likely derived from its matrix components such as fibronectin. These TEC have been shown to facilitate cartilage repair in animal models^17,18^. The repair of chondral defects with implanted TEC is now at the stage of clinical application^19,20^. As noted earlier, the TEC approach was also extended to studies of osteochondral defect repair, however, it was determined that the approach of using a TEC alone was not sufficient to completely repair large osteochondral defects in rabbit models. Therefore, a hydroxyapatite-based artificial bone was combined with a TEC in an attempt to fill large osteochondral defects. Implantation of such composite constructs demonstrated good osteochondral repair in an animal model^9^. While a promising approach, the use of an artificial scaffold does present some potential limitations and therefore, development of other innovative approaches is needed.

Based on their adhesive properties and plasticity, one innovative approach could use TEC wrapped around an iPSC-CP to form a hybrid implant comprised of two different cell types, and one that would both stabilize the implant and employ the positive features of each element. On the other hand, it is not clear whether the MSC within a TEC are needed because the adhesive properties of TEC are based on their matrix composition. Therefore, a comparison of the effectiveness of using TEC without living MSC with the original TEC containing living MSC would likely reveal the answer to this question.

The present proof of concept study aimed to investigate the effect of using hybrid implants consisting of human iPSC-CP combined with a TEC containing human MSC for biphasic osteochondral repair using a rat osteochondral defect model. While this is a xenogeneic model, it does allow for the assessment of human cells in an immunocompromised preclinical model. We hypothesized that an iPSC-CP wrapped with a basic TEC would be securely stabilized at the lesion base and to the adjacent osteochondral tissue, leading to an enhanced capability to promote osteochondral repair. It was determined that live MSC within a TEC were required to generate an effective composite hybrid implant response as using TEC with killed MSC compromised the effectiveness of the hybrid implant to facilitate defect repair. The characterization of the response to implantation of such a hybrid construct has led to the conclusion that this approach could be effective for the biphasic repair of human osteochondral defects in the future.

## Results

### Characterization of TEC, fdTEC, and iPSC-CP constructs

**Fig. 1:**
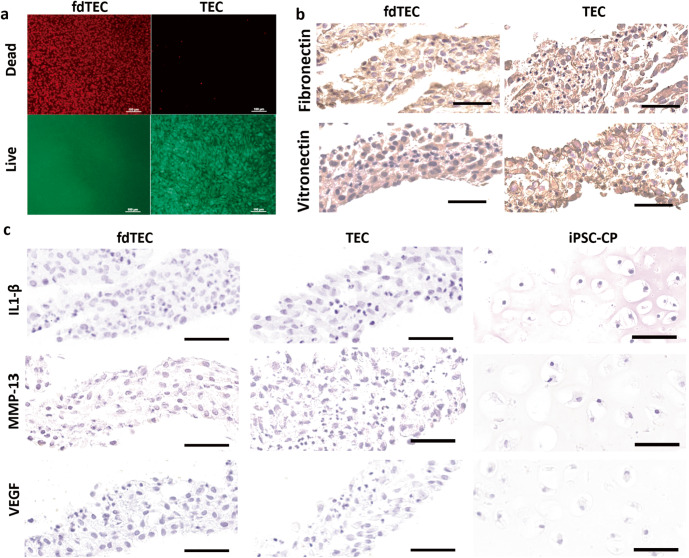
Characterization of materials. **a** Dead and Live cell viability staining of an fdTEC (left) or TEC (right). Red positive cells indicated dead cells (left; Bars = 100 μm). Green positive cells indicated live cells (right; Bar = 100 μm). **b** Immunohistochemical analysis of an fdTEC (left) and TEC (right). Bar = 50 μm. Immunohistochemical analysis for fibronectin (upper) and vitronectin (lower). **c** Immunohistochemical analysis for interleukin-1β (IL-1β: upper), matrix metalloprotease-13 (MMP-13: middle), and vascular endothelial growth factor (VEGF: lower) in an fdTEC (left), a TEC (middle), or an iPSC-CP (right). (Upper: Bar = 50 μm).

Following release from the culture dish, the layers of both the fdTEC and the basic TEC were integrated and a spontaneous series of foldings led to the development of one spherical body. Dead and live cell viability staining indicated that no live cells within the fdTEC could be detected following the freeze-drying and rehydration treatment (Fig. 1a). Both the TEC and fdTEC were diffusely stained for fibronectin and vitronectin throughout the matrix (Fig. 1b).

The original TEC, rehydrated fdTEC, and the iPSC-CP showed no detectable expression of IL-1β, MMP-13, or VEGF prior to implantation (Fig. 1c).

### Both fdTEC and basic TEC promote the initial fixation of iPSC-CP to osteochondral defects

**Fig. 2:**
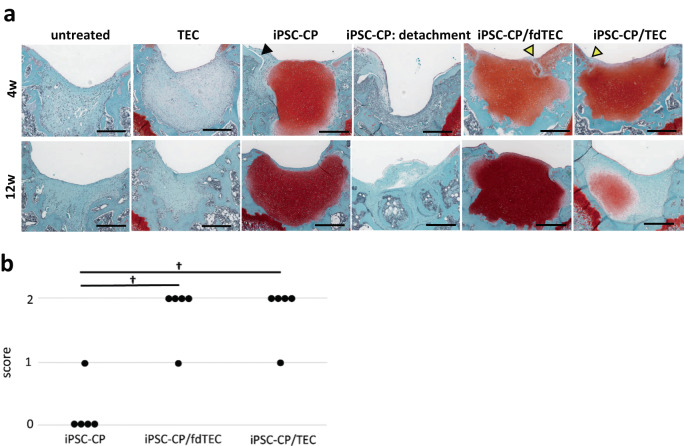
Histological images of implantation sites at 4 and 12 weeks post-operatively. **a** Histology of the tissue stained with Safranin-O (SO) at 4 weeks and 12 weeks postoperatively. Bar = 500 μm. A black arrowhead demonstrated a gap between the articular surface and the iPSC-CP. Yellow arrowheads demonstrated good integration between the iPSC-CP and the adjacent articular surface. **b** Modified O’Driscoll scores in the iPSC-CP group, the iPSC-CP/fdTEC group, and the iPSC-CP/TEC groups (*N* = 5) for bonding to adjacent cartilage at 4 weeks postoperatively. ^†^*p* = 0.007.

As shown in Fig. 2a, at 4 weeks, the defects were partially covered with granulation-like tissue in the untreated control group and the TEC only group. In contrast, the defects were consistently filled with the iPSC-CP from the level of the articular surface to the lesion base in the iPSC-CP implantation group where the implant had not detached. In some of the iPSC-CP group, the implanted iPSC-CP had detached and the defect remained empty. The engraftment of implants in the iPSC-CP group was 65.4% (4 weeks: 63.6%, 12 weeks: 62.5%, 24 weeks: 71.4%) throughout the assessment period, whereas successful implantation in the iPSC-CP/fdTEC and the iPSC-CP/TEC groups was 100%. There were gaps between the articular surface and the adjacent tissue observed in the iPSC-CP group where detachment of the iPSC-CP had not occurred. In contrast, integration to adjacent cartilage in the iPSC-CP/fdTEC or iPSC-CP/TEC group was consistently attained. Modified O’Driscoll scores in the iPSC-CP group, the iPSC-CP/fdTEC group, and the iPSC-CP/TEC group (*n* = 5) for bonding to adjacent cartilage were determined. The histological scores for bonding to adjacent cartilage were significantly higher in both the iPSC-CP/fdTEC group and the iPSC-CP/TEC group (4.5 ± 0.5) than in the iPSC-CP group (0.5 ± 0.5) (Fig. 2c).

### The presence of living cells within the TEC influences the outcome of biphasic osteochondral repair

As shown in Fig. 2a at 12 weeks post-implantation the defects remained empty in the iPSC-CP-detached cases. In contrast, the defects were consistently filled with Safranin-O stained material, which was similar in staining intensity in the iPSC-CP group, and the iPSC-CP/fdTEC group. Of relevance, the size of the safranin-O stained area and staining intensity within the iPSC-CP was reduced compared to the samples from the iPSC-CP/TEC group. At this point in time, there was no apparent subchondral repair detected in any of the groups.

**Fig. 3:**
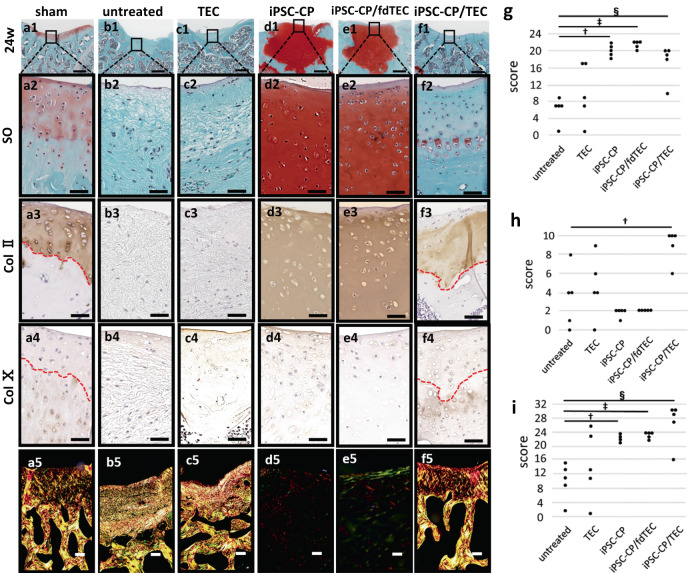
Histological images, and immunohistochemical analysis for col II or col X in implantation sites at 24 weeks postoperatively. **a1**–**f1** Histology of the tissue stained with Safranin O (SO) (Bar = 500 μm), and (**a2**–**f2**) high-magnification images of articular surface stained with SO (Bar = 100 μm). **a3**–**f4** Immunohistochemical analysis of articular surface with col II (**a3**–**f3**) and col X (**a4**–**f4**). **a5**–**f5** Polarization microscope images of the repaired tissue (Bar = 100 μm). **a3–4**, **f3–4** The red dotted lines demonstrated tidemarks. Bar = 100 μm. **g**–**i** Dot plots of modified O’Driscoll scores for cartilage repair (**g**), subchondral repair; (**h**) and the sum of cartilage and subchondral repair; (**i**) in the iPSC-CP, iPSC-CP/fdTEC group, and iPSC-CP/TCE groups at 24 weeks postoperatively. (**g**) ^†^*p* = 0.015. ^‡^*p* = 0.013. ^§^*p* = 0.014; (**h**) ^†^*p* = 0.025. **i** †: p = 0.016. ^‡^*p* = 0.015. ^§^*p* = 0.016.

At 24 weeks post-implantation, the defects in the untreated group were partially filled with fibrous tissue lacking expression of Col II (Fig. 3b1–3) as were the TEC group (Fig. 3c1–3). In contrast, the osteochondral defects were completely filled with hyaline-like cartilaginous tissue with an unchanged intensity of Safranin-O staining subsequent to implantation in the iPSC-CP (Fig. 3d1, 2) and iPSC-CP/fdTEC groups (Fig. 3e1, 2). Notably, the intensity of Safranin-O staining observed in the iPSC-CP group and the iPSC-CP/fdTEC group appeared to be somewhat higher than within normal cartilage tissue (Fig. 3f1, 2). Expression of Col II was observed coincident with the area stained with safranin-O similarly in the iPSC-CP (Fig. 3d3) and iPSC-CP/fdTEC groups (Fig. 3e3), as well as in the normal cartilage control (Fig. 3a3). There was no subchondral repair detected in the iPSC-CP and iPSC-CP/fdTEC groups. Histologically, the implanted tissue in these groups appeared to be isolated and largely unchanged from the time of implantation. In contrast, the repair tissue in the iPSC-CP/TEC group exhibited a biphasic osteochondral repair, in which the level of repair of the subchondral bone was indistinguishable from that of the adjacent undamaged tissue (Fig. 3f1, 2). Also, in samples from this group and similar to normal cartilage tissue (Fig. 3a2–4), the formation of the transition zone to the subchondral bone that included a tidemark (Fig. 3a3, f3: red dot line) and a calcified cartilaginous zone as confirmed by the positive staining of Col X (Fig 3a4, f4) could be detected. Within the cartilaginous repair tissue over the surface of the subchondral bone in the iPSC-CP/TEC group, round-shaped cells were observed in lacuna of the matrix, which was stained less intensely with safranin O than were samples from the iPSC-CP and iPSC-CP/fdTEC groups (Fig. 3f2) but were positively stained with Col II (Fig. 3f3). Observation using a polarization microscope revealed that there was no detectable subchondral repair in the samples from the iPSC-CP (Fig. 3d5) and iPSC-CP/fdTEC (Fig. 3e5) groups. In contrast, the repair tissue in the iPSC-CP/TEC group exhibited substantial biphasic osteochondral repair (Fig. 3f5), which was structurally similar to the articular surface and the subchondral area of the sham group (Fig. 3a5), but was slightly different from that of the untreated group (Fig. 3b5) and the TEC group (Fig. 3c5).

Most categories for the histological scores related to cartilage repair in the iPSC-CP, iPSC-CP/fdTEC, and iPSC-C/TEC groups were higher than the untreated defects at all time points assessed (Table 1). At 24 weeks postoperatively, the scores for safranin O staining in the iPSC-CP (2.4 ± 0.2) and iPSC-CP/fdTEC (2.4 ± 0.2) groups were significantly higher than those for the untreated group, but not for the iPSC-CP/TEC group (1.4 ± 0.5). Total histological scores for cartilage repair were significantly higher in the iPSC-CP (20.0 ± 0.7) and iPSC-CP/fdTEC (21.4 ± 0.4), and iPSC-CP/TEC group (17.4 ± 1.9) than in the untreated group (6.2 ± 1.4) (Fig. 3g). In the case of the subchondral repair, most categories of the histological scores for subchondral repair in the iPSC-CP/TEC group increased over time, while those in the iPSC-CP and iPSC-CP/fdTEC group remained low without detectable change (Table 2). At 24 weeks postoperatively, total histological scores for subchondral repair were highest in the iPSC-CP/TEC group (9.0 ± 1.7) compared to the untreated group (3.4 ± 1.4) (Fig. 3h). The sums of total histological scores for cartilage and subchondral repair were significantly higher in the iPSC-CP (22.0 ± 0.7), iPSC-CP/fdTEC (23.4 ± 0.4), and iPSC-CP/TEC (26.4 ± 2.7) groups than for the untreated group (9.6 + 2.3) (Fig. 3i).

**Table 1:** Histological scores for cartilage repair using the modified O’Driscoll scoring system^19,41^ at 4 weeks, 12 weeks, and 24 weeks postoperatively.

		untreated	TEC	iPSC-CP	iPSC-CP/fdTEC	iPSC-CP/TEC
Cellular morphology [0–4]	4w	0	0	3.8 ± 0.3**	4 ± 0**	3.8 ± 0.2**
	12w	0.6 ± 0.3	1.2 ± 0.2	3.4 ± 0.4*	4 ± 0**	2.4 ± 0.5*
	24w	1.2 ± 0.37	1.8 ± 0.5	3.6 ± 0.2*	3.8 ± 0.2*	2.8 ± 0.2*
Safranin O staining [0–3]	4w	0	0	2.6 ± 0.3**	2.8 ± 0.2**	2.6 ± 0.2**
	12w	0.4 ± 0.3	0.4 ± 0.2	2.2 ± 0.4*	3 ± 0**	1.8 ± 0.4*
	24w	0.2 ± 0.2	0.4 ± 0.2	2.4 ± 0.2*	2.4 ± 0.2**	1.4 ± 0.5
Surface regularity [0–3]	4w	0.2 ± 0.2	0.2 ± 0.2	2.6 ± 0.3*	2.8 ± 0.2*	2.8 ± 0.2*
	12w	0.4 ± 0.3	1.0 ± 0.5	2.2 ± 0.2*	3 ± 0**	1.8 ± 0.2*
	24w	0.8 ± 0.2	1.2 ± 0.4	2.4 ± 0.2*	3.0 ± 0*	2.6 ± 0.4
Structural integrity [0–2]	4w	0.2 ± 0.2	0.2 ± 0.2	1.2 ± 0.2	1.8 ± 0.2*	1.8 ± 0.2*
	12w	0.4 ± 0.3	1.2 ± 0.4	2 ± 0**	1.8 ± 0.2*	1.8 ± 0.2*
	24w	0.8 ± 0.2	1.0 ± 0.3	1.6 ± 0.2	2.0 ± 0**	2.0 ± 0**
Thickness [0–2]	4w	0	0	2 ± 0**	2 ± 0**	2 ± 0**
	12w	0.4 ± 0.3	1.0 ± 0.5	1.8 ± 0.2*	2 ± 0**	1.8 ± 0.2*
	24w	0.6 ± 0.6	1.2 ± 0.4	2.0 ± 0**	2.0 ± 0**	1.6 ± 0.3
Bonding to adjacent cartilage [0–2]	4w	0	0	0.2 ± 0.2	1.8 ± 0.2**	1.8 ± 0.2**
	12w	1.0 ± 0.3	1.0 ± 0.5	2 ± 0*	1.6 ± 0.2	1.8 ± 0.2
	24w	1.0 ± 0.3	1.2 ± 0.4	1.6 ± 0.2	1.8 ± 0.2	2.0 ± 0*
Hypocellularity [0–3]	4w	0.8 ± 0.2	0.8 ± 0.2	3 ± 0**	3 ± 0**	3 ± 0**
	12w	0.4 ± 0.3	1.4 ± 0.6	2.6 ± 0.2	3 ± 0*	2 ± 0.3
	24w	0.8 ± 0.2	1.6 ± 0.4	3.0 ± 0**	3 ± 0**	2.4 ± 0.4*
Chondrocyte clustering [0–2]	4w	0	0.2 ± 0.2	2 ± 0**	2 ± 0**	2 ± 0**
	12w	0.2 ± 0.2	1.0 ± 0.5	1.8 ± 0.2*	2 ± 0**	1 ± 0.3
	24w	0.2 ± 0.2	1.2 ± 0.4	2.0 ± 0**	2 ± 0**	1.8 ± 0.2*
Degeneration of adjacent cartilage [0–3]	4w	1.2 ± 0.2	1.6 ± 0.2	1.6 ± 0.2	1.6 ± 0.2	1 ± 0
	12w	0.8 ± 0.2	0.6 ± 0.4	1.4 ± 0.2	2 ± 0**	1.4 ± 0.2
	24w	0.6 ± 0.4	0.6 ± 0.2	1.4 ± 0.2	1.4 ± 0.2	0.8 ± 0.2
Total score [0–24]	4w	2.4 ± 0.4	3.0 ± 0.5	19 ± 0.6*	21.8 ± 0.5*	20.8 ± 0.5*
	12w	4.4 ± 0.9	8.8 ± 3.1	19.4 ± 1.5*	22.4 ± 0.4*	15.8 ± 1.2*
	24w	6.2 ± 1.4	10.2 ± 3.0	20.0 ± 0.7*	21.4 ± 0.4*	17.4 ± 1.9*

Compared to untreated group. *P < 0.05, **P < 0.01.

**Table 2:** Histological scores for subchondral repair using the modified O’Driscoll scoring system^19,41^ at 4 weeks, 12 weeks, and 24 weeks postoperatively.

		untreated	TEC	iPSC-CP	iPSC-CP/fdTEC	iPSC-CP/TEC
Subchondral bone alignment [0–2]	4w	0	0	0	0	0
	12w	0.2 ± 0.2	0.4 ± 0.2	0	0	0.2 ± 0.2
	24w	0.8 ± 0.4	1.2 ± 0.4	0	0	1.8 ± 0.2
Bone infiltration into defect area [0–2]	4w	0	0	0	0	0
	12w	0.6 ± 0.2	0.8 ± 0.5	0	0	0.2 ± 0.2
	24w	0.8 ± 0.4	1.0 ± 0.3	0	0	1.8 ± 0.2
Tidemark continuity [0–2]	4w	0	0	0	0	0
	12w	0.2 ± 0.2	0.4 ± 0.2	0	0	0
	24w	0.2 ± 0.2	0.4 ± 0.2	0	0	1.4 ± 0.4
Cellular morphology [0–2]	4w	0	0	0	0	0
	12w	0.8 ± 0.2	0.8 ± 0.2	0*	0*	0.2 ± 0.2
	24w	0.8 ± 0.2	1.0 ± 0.3	0	0	2.0 ± 0**
Exposure of subchondral bone [0–2]	4w	0.2 ± 0.2	0.2 ± 0.2	2.0 ± 0*	2.0 ± 0*	2.0 ± 0*
	12w	0.4 ± 0.2	0.8 ± 0.4	2.0 ± 0*	2.0 ± 0*	2.0 ± 0*
	24w	0.8 ± 0.4	1.0 ± 0.3	2.0 ± 0*	2.0 ± 0*	2.0 ± 0*
Total score [0–12]	4w	0.2 ± 0.2	0.2 ± 0.2	2.0 ± 0*	2.0 ± 0*	2.0 ± 0*
	12w	2.2 ± 0.9	3.2 ± 0.4	2.0 ± 0	2.0 ± 0	2.6 ± 0.6
	24w	3.4 ± 1.4	4.6 ± 1.5	2.0 ± 0	2.0 ± 0	9.0 ± 1.7*

Compared to untreated group. *P < 0.05, **P < 0.01.

### Radiological assessment for evaluation of subchondral bone repair

**Fig. 4:**
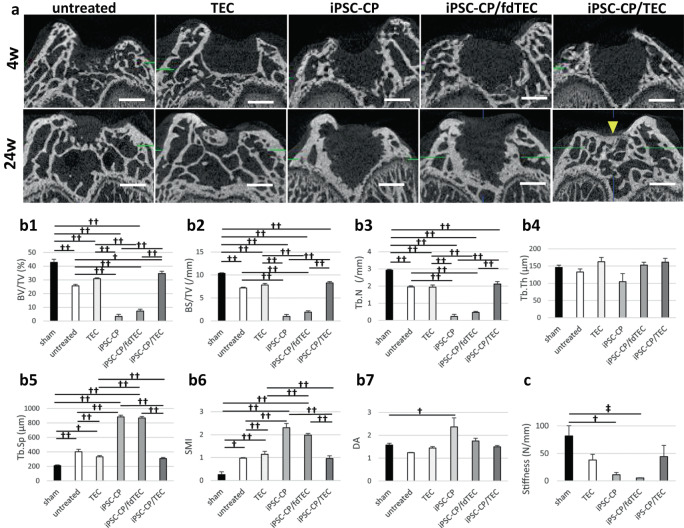
Micro-CT analysis and mechanical analysis for osteochondral bone and cartilage. **a** Micro-CT image of five groups at 4 (upper) and 24 weeks (lower) postoperatively. Bar = 1 mm. **b1–5** Quantification of BV/TV (%; **b1**), BS/TV (/mm; **b2**), Tb.N (/mm; **b3**), Tb.Th (μm; **b4**), Tb.Sp (μm; **b5**), SMI (**b6**), and DA (**b7**) at 24 weeks postoperatively and sham samples of 34 weekly aged rats (*N* = 5). ^†^*p* < 0.01, ^††^*p* < 0.001. **c** The stiffness of the sham group (*N* = 5), the TEC group (*N* = 5), the iPSC-CP group (*N* = 5), the iPSC-CP/fdTEC group (*N* = 5), and the iPSC-CP/TEC group (*N* = 5). ^†^*p* = 0.015, ^‡^*p* = 0.008; data represent mean ± SD.

Subchondral repair was also evaluated using micro-computed tomography (Fig. 4a). In the untreated defect group, newly formed bone was first detected at 4 weeks and increased over time until 24 weeks post-injury. However, the untreated defects failed to restore the subchondral bone to the levels of normal tissue. In the TEC group, newly formed bone was not detected at 4 weeks but was partially detected at 24 weeks. Conversely, bone formation was not observed in the defects implanted with an iPSC-CP or an iPSC-CP/fdTEC construct up to 24 weeks post-implantation. In the iPSC-CP/TEC group, bone formation was not detected at 4 weeks, but hereafter, bone formation proceeded over time and complete repair of the subchondral bone was detected by 24 weeks. The surface level of the bone was also equivalent to that of adjacent tissue at 24 weeks. At 24 weeks, the BV/TV of defect space in the iPSC-CP/TEC group (34.7 ± 5.0%) was significantly larger than the iPSC-CP group (4.7 ± 2.5%: *p* < 0.001) or the iPSC-CP/fdTEC group (6.7 ± 1.5%: *p* < 0.001) (Fig. 4b1). The BS/TV and Tb.N of the defect spaces in the iPSC-CP/TEC group (BS/TV: 8.7 ± 0.8 /mm, Tb.N: 2.1 ± 0.1 /mm) were significantly larger than the same parameters of the iPSC-CP group (BS/TV: 1.5 ± 0.6 /mm; *p* < 0.001, Tb.N: 0.2 ± 0.1 /mm; *p* < 0.001) or the iPSC-CP/fdTEC group (BS/TV: 1.5 ± 0.7 /mm; *p* < 0.001, Tb.N: 0.5 ± 0.1 /mm; *p* < 0.001) (Fig. 4b2–3). The trend of the BV/TV was similar to the BS/TV and the Tb.N. There was no significant difference in the Tb.Th between the groups (Fig. 4b4). The Tb.Sp of the defect space in the iPSC-CP/TEC group (0.3 ± 0.0 mm) was significantly smaller than the same parameter of the iPSC-CP group (0.9 ± 0.0 mm: *p* < 0.001) or the iPSC-CP/fdTEC group (0.9 ± 0.0 mm: *p* < 0.001) (Fig. 4b5). The trend of the Tb.Sp was similar to the SMI. The SMI and the DA between the sham and the iPSC-CP/TEC group were not significantly different (Fig. 4b6–7).

### Mechanical properties of the repair tissue following implantation of cell constructs

The stiffness of the repair tissue was assessed using indentation testing of the cartilage-subchondral bone complex specimens. The untreated defect group and the TEC group which did not form cartilaginous repair tissue were excluded from the analysis. At 24 weeks post-implantation, the mean stiffness of the repair tissue in the iPSC-CP group (11.0 ± 4.4 N/mm: *p* = 0.015) and the iPSC-CP/fdTEC group (5.2 ± 0.6 N/mm: *p* = 0.008) was significantly lower than that for normal cartilage (81.8 ± 18.6 N/mm). In contrast, there was no statistically significant difference in the stiffness of the repair tissue in the samples from the iPSC-CP/TEC group (44.3 ± 20.6 N/mm: *p* = 0.464) compared to normal cartilage although the outcomes were variable between animals (Fig. 4c).

### Assessment of the fate of the implanted cells

**Fig. 5:**
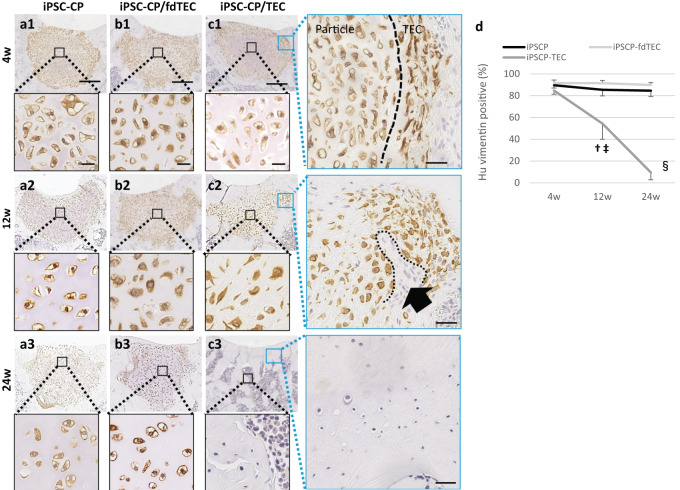
Immunohistochemical analysis for human vimentin to detect the origin of the cells in repair tissue following implantation of the three constructs. Immunohistochemical analysis for human vimentin in the iPSC-CP group (**a1**–**3**) and the iPSC-CP/fdTEC group (**b1**–**3**), and the iPSC-CP/TEC group (**c1**–**3**) at 4, 12, and 24 weeks postoperatively (left; Bar = 500 μm). High-magnification images of central iPSC-CP in the iPSC-CP group, the iPSC-CP/fdTEC group, and the iPSC-CP/TEC group (lower; black square, Bar = 20 μm). **c** High-magnification images of the border area between the iPSC-CP and host tissue (blue square; left) in the iPSC-CP/TEC group. Bar = 100 μm. Black arrow demonstrated that the host cells invaded into an iPSC-CP. The black dotted line indicated the border between the host cells and the iPSC-CP. **d** Quantification of human vimentin positive percentage in the iPSC-CP group (*N* = 5), the iPSC-CP/fdTEC group (*N* = 5), and the iPSC-CP/TEC group (*N* = 5) at 4, 12, and 24 weeks postoperatively. ^†^*p* = 0.005 (compared to the iPSC-CP/fdTEC group), ^‡^*p* = 0.029 (compared to the iPSC-CP group), ^§^*p* < 0.001 (compared to the iPSC-CP group and the iPSC-CP/fdTEC group); data represent mean ± SD.

To identify the fate of the cells in the implanted constructs, the cells were labeled with human vimentin and the distribution of the labeled cells was followed over time post-implantation. The majority of the implanted human cells were round-shaped and remained populated within the implanted tissue in the iPSC-CP (Fig. 5a1–3) and the iPSC-CP/fdTEC groups (Fig. 5b1–3) until 24 weeks. In the iPSC-CP/TEC group, round-shaped human cartilaginous cells predominantly populated the implanted tissue at 4 weeks, with a clear border between round shape cells and spindle-shaped cells being recognized (Fig. 5c1). Thereafter, spindle-shaped human cells were observed in the center area of the implanted tissue, some of which were human vimentin negative at 12 weeks (Fig. 5c2). The percentage of human vimentin positive cells in the implanted tissue in the iPSC-CP/TEC group decreased over time. By 24 weeks, no cells within the repair tissue in the iPSC-CP/TEC group were human vimentin positive including newly repaired cartilage and bone (Fig. 5c3). In contrast, the percentage of vimentin positive human cells within the implanted tissue in the group receiving an iPSC-CP alone or receiving an fdTEC was maintained at a high level to 24 weeks in the group receiving an iPSC-CP alone (Fig. 5d).

### IL-1β, MMP-13, and VEGF expression and subchondral repair

To assess the involvement of potential endochondral ossification mechanisms associated with the subchondral bone repair, expression of interleukin-1β (IL-1β), matrix metalloprotease-13 (MMP-13), and vascular endothelial growth factor (VEGF) were evaluated by immunohistochemistry. The original TEC, rehydrated fdTEC, and the iPSC-CP showed no detectable expression of IL-1β, MMP-13, or VEGF prior to implantation (Fig. 1c).

**Fig. 6:**
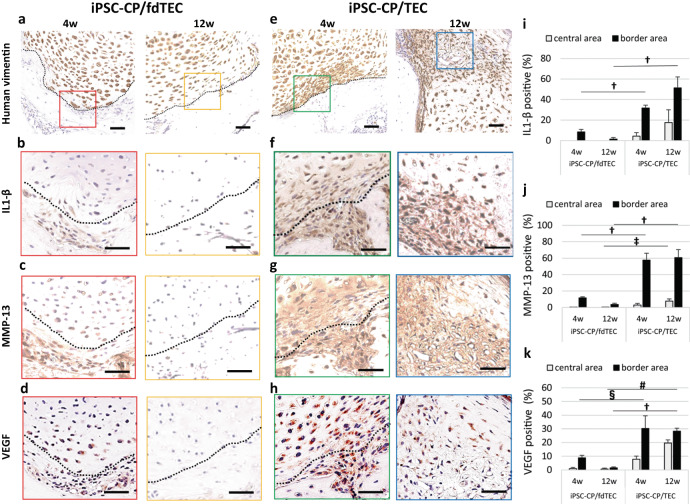
Immunohistochemical analysis for human vimentin, IL-1β, VEGF, and MMP-13 at the border area between implanted materials and host tissue. **a**, **e** Immunohistochemical analysis for human vimentin in the iPSC-CP/fdTEC group and in the iPSC-CP/TEC group. Bar = 50 μm. High-magnification images of color rectangle: Immunohistochemical analysis for IL-1β (**b**, **f**), MMP-13 (**c**, **g**), and VEGF (**d**, **h**) in the iPSC-CP/fdTEC group (**a–d**) and the iPSC-CP/TEC group (**e**–**h**). Bar = 50 μm. **i**–**k** Quantification of IL-1β (**i**), MMP-13 (**j**), and VEGF (**k**) positive percentage in the iPSC-CP/fdTEC group (*N* = 5) and the iPSC-CP/TEC group (*N* = 5) at 4 and 12 weeks postoperatively. ^†^*p* < 0.001, ^‡^*p* = 0.026, ^§^*p* = 0.030, ^#^*p* = 0.006; data represent mean ± SD.

However, following implantation, in the iPSC-CP/fdTEC group, the boundary between the implanted iPSC-CP and the adjacent host tissue was clearly observed as confirmed by human vimentin staining (Fig. 6a). IL-1β, MMP-13, and VEGF were detected around the adjacent host cells at 4 weeks, but expression levels were clearly reduced by 12 weeks (Fig. 6b–d). Conversely, in the iPSC-CP/TEC group, IL-1β, MMP-13, and VEGF were intensely expressed around the adjacent host cells and the cells located at the periphery of implanted tissue as confirmed by human vimentin staining (Fig. 6e–h; left) at 4 weeks post-implantation. At 12 weeks post-implantation, IL-1β, MMP-13, and VEGF were detected throughout the matrix of the implanted tissue in the samples implanted with an iPSC-CP (Fig. 6e–h; right). The percentage of positive cells stained for IL-1β, MMP-13, and VEGF in the iPSC-CP/TEC group increased in a time-dependent manner for both the central and border areas, and the percentages were significantly higher than those in the iPSC-CP/fdTEC group at 12 weeks (Fig. 6i–k).

### Assessment of angiogenesis and new blood vessel formation into the iPSC-CP in the iPSC-CP/TEC group following implantation

**Fig. 7:**
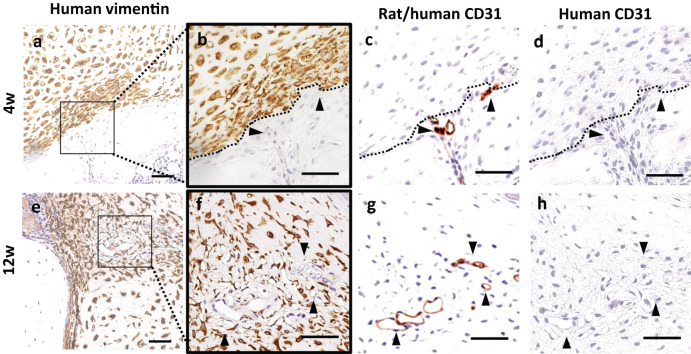
Immunohistochemical analysis for human vimentin, rat/human CD31, and human CD31 at the border area between implanted materials and host tissue. Immunohistochemical analysis for human vimentin (**a**, **e**) and high magnification for human vimentin (**b**, **f**), human/rat CD31 (**c**, **g**) and human CD31 (**d**, **h**) at 4 weeks (**a**–**d**) and 12 weeks (**e**–**f**) in the iPSC-CP/TEC group. **a**–**h** Bar = 50 μm. **b**–**d**, **f**–**h** Black dotted lines indicated the border between the host tissue and the iPSC-CP. Black arrows indicated the vessels.

At 4 weeks post-implantation, new blood vessels were observed in the peripheral area of the implanted tissue (Fig. 7a, b) that stained positive with a rat/human CD31 (Fig. 7c) but were negative for human CD31 staining (Fig. 7d), suggesting that new blood vessels were derived from the host rat cells. At 12 weeks, new blood vessels that were stained with a rat/human CD31 (Fig. 7g) but were negative for human CD31 staining (Fig. 7h) were observed inside the implanted iPSC-CP where some cells were also human vimentin negative (Fig. 7e, f).

## Discussion

There are a number of important conclusions that evolve from the studies presented. The first is that wrapping or encasing an iPSC-CP containing human differentiated induced pluripotent stem cells with a tissue engineered construct (TEC) derived from human synovial MSC leads to the stable incorporation of the hybrid construct compared to implanting an iPSC-CP alone into osteochondral defects in a rat knee. Secondly, it does not matter whether live cells are present in the TEC to foster stable implantation of the hybrid. Thirdly, in contrast to the stability of the implantation, live cells are required in the TEC to facilitate the successful development of a bi-layered tissue to repair the osteochondral defects with the iPSC-CP. Fourthly, neither implanted human iPSC nor MSC in the hybrid iPSC-CP/TEC constructs could be detected in the repair tissue by 24 weeks post-implantation and thus, infiltration of the constructs by host cells occurs over time post-implantation.

Thus, in the hybrid constructs, the TEC containing live MSC appear to serve two important functions in these composite implants, the non-augmented adhesion of the hybrid TEC-iPSC-CP to the lesion site and then subsequently, the enhancement of the repair process with the development of a biphasic repair tissue containing host cells by 24 weeks post-implantation.

The present studies also demonstrated that the TEC containing live MSC or fdTEC containing dead MSC similarly promoted the initial stabilization of iPSC-CP in osteochondral defects with secure integration to adjacent host tissue following implantation. This finding suggests that the adhesive properties of matrix components of the TEC have contributed to the secure stabilization of iPSC-CP into the osteochondral defect. However, unexpectedly, the choice of whether the TEC containing live MSC or fdTEC with dead MSC was used to form the hybrid constructs leads to very different outcomes regarding osteochondral repair. While the use of a rehydrated fdTEC for the hybrid constructs likely helped stabilization of iPSC-CP to the lesion site, the overall repair process was similar to that of using an iPSC-CP alone. Interestingly, both the iPSC-CP/fdTEC and iPSC-CP groups failed to repair of the subchondral bone but the implanted iPSC-CP in these groups appeared to maintain their hyaline cartilaginous matrix and the original cell population as if acting as an isolated island of cells. In contrast, the iPSC-CP/TEC group showed a dynamic conversion from the original hyaline cartilaginous matrix to a biphasic osteochondral tissue. Notably, the cells that repopulated the repair tissue were host (rat) cells within 24 weeks and thus, the human cells in the original implants were replaced by an unknown mechanism as these rats were immunocompromised.

Previous studies have suggested that the process of subchondral bone repair using in vitro generated chondrogenic masses derived from MSC may have an analogy with endochondral ossification using various primers^21–23^. In such studies, in vitro generated chondrogenic masses from MSC required IL-1β as a primer to be efficiently remodeled into the bone and bone marrow paralleled by ingrowth of blood vessels. Thus, we hypothesized as to whether similar mechanisms could also be involved in the present studies, and therefore investigated the expression patterns for IL-1β, MMP-13, and VEGF in samples from the various implants. In the iPSC-CP/TEC group, expression of these molecules was localized around the peripheral margin of the implanted iPSC-CP at 4 weeks but by 24 weeks was detected throughout the matrix of the iPSC-CP and paralleled the invasion of host (rat) cells. Interestingly, in the iPSC-CP/fdTEC group, expression of IL-1β, MMP-13 and VEGF were likewise detected around the periphery of the iPSC-CP but hereafter the expression was barely detected within or around the implanted iPSC-CP, even though the hyaline cartilaginous matrix was maintained until 24 weeks. This finding was paralleled with the failure of host (rat) cells to invade into the matrix.

Taken together, implanted iPSC-CP maintained their matrix composition, as well as their cellular population throughout the observation period as if by rejecting cellular communication between the implanted cells and host cells when delivered alone or wrapped with an fdTEC containing no viable cells. Interestingly, the cells and the matrix within the iPSC-CP were maintained throughout the observation period of 24 weeks post-implantation in this scenario. Thus, host cells were not able to penetrate this “island” of iPSC in a proteoglycan-rich matrix to modify the matrix or the cells (as was the case with the basic TEC/iPSC-CP hybrid implants). While not the focus of the present studies, future elucidation of the mechanisms involved in such resistance by the iPSC-CP may provide important clues as to the regulation of articular cartilage and maintenance of the cellular integrity of the tissue.

Conversely, the implanted iPSC-CP was totally replaced by biphasic osteochondral tissue when wrapped with a basic TEC containing live MSC. Such differences clearly indicate that the presence of living MSC within the TEC presumably have an important role in controlling and regulating the signals for subchondral bone repair including host cellular invasion into the chondrogenic mass and vascular ingrowth. This may be the first evidence showing the significance of MSC to promote endochondral ossification in vivo as indicated by the comparison of the outcomes of the studies with the hybrid constructs prepared with a basic TEC or one that had been freeze-dried to kill the cells. Many tissue-engineered cartilaginous particles derived from various cells have been developed, however, their fixation to the implanted site was one of the issues in this field and suturing with biocompatible threads or fixation with materials such as fibrin glue were examined. The basic TEC containing live MSC may resolve this issue as the adhesive reinforcement materials for other tissue-engineered cartilaginous particle, and moreover, may achieve biphasic repair similar to this study.

Mechanistically, it is not known from these proof-of-principle studies presented regarding how the cells communicate or what vehicles they use to communicate. However, it is well known that both MSC and iPSC can release metabolically active signaling molecules including those in exosomes and extracellular vesicles^24–28^, so they may also use those mechanisms to communicate between themselves and with host cells. Some of these options could be explored in future studies in an in vitro setting using secreted molecules and exosomes/extracellular vesicles from the different cell populations to assess their impact on either host cells, or their counterparts in such hybrid constructs.

Additional insights into the possible mechanisms involved in the successful development of biphasic repair tissue (e.g., the iPSC-CP/TEC group) can be surmised from those groups where this did not occur (e.g., the iPSC-CP alone and iPSC-CP/fdTEC group). The failure of the latter may be due to the presence of matrix molecules in the cartilage particles that arise during differentiation of the original iPSC that are anti-angiogenic, or even due to molecules secreted by the differentiated cells. Articular cartilage is known to exhibit anti-angiogenic effects and loss of such properties may play a role in the process of osteoarthritis^29–32^. Thus, in the situation where an iPSC-CP was implanted in the absence of viable cells in a TEC, the cartilage particles resisted migration of host cells into the tissue, potentially due to the development of a cartilage-like matrix and with cells secreting cartilage-associated molecules. Therefore, when MSC are present in a hybrid implant, these cells appear to either alter the molecular expression phenotype of the iPSC-CP cells or induce the degradation of anti-neovascularization matrix molecules, either via the release of exosomes or secretion of molecules from the viable MSC in the TEC. Studies to discern some of the above discussed alternatives are feasible and will be the subject of future investigations.

Finally, by 24 weeks post-implantation with a hybrid construct with an iPSC-CP wrapped in a basic TEC, no human cells could be detected in the repair tissue, only host cells. This finding is consistent with other studies where the implanted cells disappeared over time even in a human into human scenario^33^. Why and how the implanted cells die and are removed once they appear to have stimulated host cells appropriately is not known. Interestingly, the cells in the iPSC-CP were still present when there was no invasion of host cells, but they did disappear when host cells migrated into and repopulated the repair tissue, and thus, this may mean that the host cells somehow mediate the removal of the implanted cells via currently unknown mechanisms.

Regarding the clinical relevance of the proof-of-principle studies presented, it is clear that repair of osteochondral lesions with a well-defined biphasic repair tissue structure including both the cartilaginous and bony layers is critical to the functioning of the repair tissue. It has been reported that the biphasic structure of articular cartilage and bony foundations make a highly resilient and biomechanically efficient tissue, and both the development and maturation of osteochondral tissue facilitate functional durability^34^. On the other hand, failed repair of subchondral bone results in softening of the osteochondral complex at the repair site. Such tissue softening predisposes the tissue to loads that may contribute to the accelerated risk of cartilage degeneration and the initiation or progression towards osteoarthritis of the whole compartment^35^. In addition, many tissue-engineered cartilaginous constructs derived from various cells has been developed, however, their fixation to the implant site was one of the issues in this field and suturing with biocompatible threads or fixation with materials such as fibrin glue were examined. The basic TEC containing live MSC may resolve this issue as the adhesive reinforcement materials for other tissue-engineered cartilaginous units, and moreover, may achieve biphasic repair of defects similar to the outcomes of the present study.

There are some limitations to the present proof-of-principle studies. First, the quality of cartilaginous repair tissue in the iPSC-CP/TEC group was not comparable to normal cartilage by 24 weeks post-implantation. Although the repair tissue contained hyaline-like cartilaginous tissue with positive collagen II expression, the staining intensity of the tissue with Safranin O was weaker than that of normal cartilage. However, it should also be noted that the intensity of the repair tissue in the iPSC-CP group and the iPSC-CP/fdTEC was conversely stronger than that of normal cartilage, indicating that the quality of such repair tissue was also different from normal cartilage. This is why we also comprehensively assessed the quality of repair tissue by adding the biomechanical analysis. Careful longer-term follow-up will be required to determine whether the repair tissue continues to mature and develop characteristics closer to the properties of normal tissues. Importantly, the molecular mechanisms underlying the interaction between MSC and iPSCP, as well as these implanted cells and host cells remains unclear. Specifically, it would be of interest to elucidate the barrier mechanisms within iPSC-CP to prevent cellular invasion by host cells and how the presence of MSC leads to the overcoming of such barrier mechanisms. Future studies are needed to answer such questions or address such issues. Finally, the model used for the studies was a small immune-deficient animal model and it is not known how a model with a competent immune system could impact the outcomes. However, due to the potential adverse effects of an intact immune system, it is likely not realistic to use large animals due to the issue of tissue rejection in a xenogeneic model. Thus, for larger animal studies one may have to use homologous cell populations for the iPSC-CP and TEC as a precursor to any potential use in patients. Finally, these proof-of-principle studies were performed with constructs developed with a single human iPSC. Thus, additional research with other human iPSC derived from different cells and from individuals with different genetic backgrounds will be required to define the most optimal cells to facilitate osteochondral repair. Such studies will also serve a scientific purpose in that comparisons between iPSC cells on outcomes will provide insights into the regulation of many of the features detected in the present studies.

The studies presented have identified some important relationships between iPSC in a CP with host cells, as well as the importance of using a hybrid construct containing a TEC containing live MSC plus an iPSC-CP to facilitate the effective development of a biphasic tissue to repair osteochondral defects. These unique findings also support the possibility that such hybrid implants may also further the effectiveness of TEC to facilitate the repair of chondral defects^10,15^, particularly in the future when repair of defects associated with early osteoarthritis is attempted. Additional research with other human iPSC will potentially confirm this innovative approach to facilitate damaged cartilage and bone, as well as clinical applications to optimize the healing of other connective tissue injuries. While this line of investigation is complex, it may have significant clinical relevance going forward regarding the repair of large osteochondral defects, which unless effectively repaired/regenerated would very likely progress to overt osteoarthritis and potentially, the need for a total knee replacement.

## Methods

### Ethics statement

All procedures involving human participants were in accordance with the ethical standards of the institutional research committee in our institutions (the ethical committee of Osaka University Graduate School of Medicine, reference number: 14102-8, 16345-3) and the 1964 Helsinki declaration and its later amendments or comparable ethical standards. Written informed consent was obtained from each donor. Animal experiments were conducted after approval from the Ethics Committee of Osaka University (approval number: 01-074-001).

### Cell culture

Human synovium MSC were isolated and expanded using serum-free culture medium STK1^®^ and STK2^®^ (Kanto Chemical Co., Inc., Tokyo, Japan). Briefly, synovium was obtained from a patient with anterior cruciate ligament injury (15-year-old female) at the time of arthroscopic surgery. The synovial specimens were minced and plated in an STK1 medium. Outgrown cells from tissue fragments were cultured in STK2 for cell expansion. The cells were confirmed to be CD13, CD44, CD34, CD73, and CD90 positive by flow cytometry analysis, and their potency in chondrogenic, osteogenic, and adipogenic differentiation was validated^36–39^. The cells were defined as synovial mesenchymal stromal cells (MSC) and used at passage 5 for subsequent experiments.

### Development of the basic tissue engineered construct (TEC)

**Fig. 8:**
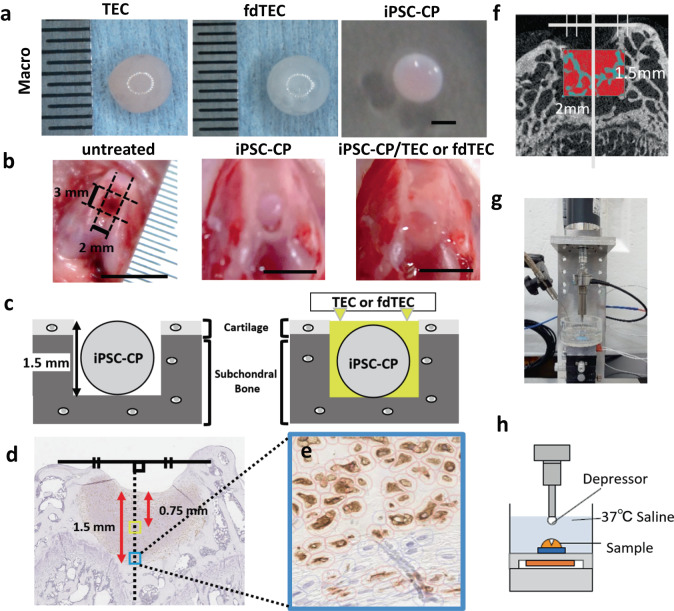
Materials and in vivo methods. **a** Macroscopic view of a TEC (left), an fdTEC (middle), and an iPSC-CP (right; Bars = 1 mm). **b** Macroscopic view of osteochondral defects in the femoral groove of the rat (left; Bar = 5 mm), an iPSC-CP group (middle; Bar = 5 mm), and an iPSC-CP/TEC or fdTEC group (right; Bar = 5 mm). **c** Schematic representation of an iPSC-CP (left) and an iPSC-CP/fdTEC or an iPSC-CP/TEC (right). **d** The region of interest (ROI) for measurement of DAB-stained positive ratio. The dotted line denotes the middle of the defect space. The yellow and green square with a 100 µm long side was extracted as the ROI of the central area and border area, respectively. **e** The high magnification image in the ROI for measurement of DAB-stained positive ratio. **f** The region of interest for bone volume measurement was shown (red rectangle). The white line denotes the middle of the defect space. **g**, **h** Picture of mechanical testing machine (**g**) and its schematic representation (**h**).

For the preparation of a basic TEC, MSC were plated on 24-well multi-well culture plates (BD Falcon science, NJ, USA) at a density of 4.0 × 10^5^ cells/cm^2^ (8.0 × 10^5^ cells/well) in growth medium containing 0.2 mM ascorbate-2-phosphate (Asc-2P), an optimal concentration from earlier studies^15,16^. After an additional culture duration of 7 days, a monolayer complex of the cultured cells with an extracellular matrix was developed, and this complex formed a three-dimensional structure by detaching from the substratum of the plate. This construct was termed the basic TEC (Fig. 8a).

### Development of a TEC containing dead MSC (fdTEC)

Basic TECs were freeze-dried for 24 h by vacuum lyophilization (Free Zone 2.5, Labconco, MO, USA). This process led to the death of the MSC and the resulting complex was termed an fdTEC (Fig. 8a). Prior to use in experiments, the fdTEC were rehydrated in sterile phosphate-buffered saline (pH 7.2, PBS) at room temperature.

### Development of iPSC-CP by chondrogenic differentiation of hiPSC OHJ1 cells

The hiPSC line QHJl was generated by reprogramming human periphera1 blood monocytes derived from a healthy male individual with homozygosity for major HLA loci and was a gift from CiRA (Kyoto University; Kyoto, Japan). The cell line was generated by electroporating episomal plasmid vectors (pCXLE-hOCT3/ 4-shp53-F, hSK, hUL, EBNA1) into the cells of origin. The hiPS OHJ1 cells were chondrogenically differentiated to produce iPSC-CP using a previously described method^14^. The iPSC-CPs were generated by chondrogenic differentiation of the cells for 10 weeks prior to being used in the current experiments (Fig. 8a). The cells in the iPSC-CP expressed collagen II as determined by qPCR (Supplementary Fig. 1), a finding that is consistent with previous reports with similar iPSC-CP^40^.

### Implantation of the combined implants to osteochondral defects

Ten-week-old male athymic nude rats (F344 NJcl-rnu/rnu; CLEA Japan, Fujinomiya, Japan) were anesthetized by an intramuscular injection of a mixture of 0.3 mg/kg of medetomidine, 4.0 mg/kg of midazolam, and 5.0 mg/kg of butorphanol. After shaving, disinfection, and draping, a straight 1 cm long medial parapatellar incision was made at both knees of each animal. One knee was used for histological and radiological analysis, and the other was used for biomechanical analysis. The patella was gently dislocated laterally and the femoral groove was exposed. Full-thickness articular osteochondral defect, which surface was a rounded rectangle with side lengths of 3 mm and 2 mm, and depth from the surface of the defect was 1.5 mm, was created in the femoral groove of the distal femur using a drill with a sphere top of 1.5 mm diameter (Fig. 8b). The defects were generated at a moderate drill speed while irrigating the site with a room temperature saline solution to prevent thermal damage to the surrounding bone and cartilage.

Seventy-five rats were divided into five groups. For the untreated control group, the defects were left empty, while for the TEC group, a basic TEC was implanted into the defect. For the iPSC-CP group, an iPSC-CP was implanted into the defects. For the preparation of the iPSC-CP/fdTEC and iPSC-CP/TEC group, an iPSC-CP was placed on the center of either a rehydrated fdTEC (iPSC-CP/fdTEC group) or a basic TEC (iPSC-CP/TEC group) whose wrinkles were smoothed out, completely wrapped with it immediately before implantation, and then these hybrid materials were implanted into the defects (Figs. 8b, c). Randomly selected rats in the five groups were euthanized at 4, 12, and 24 weeks postoperatively with carbon dioxide after anaesthetized, and the implants were assessed.

Moreover, three animals were added for specimens in the iPSC-CP group at 4 weeks, two animals were added at 12 weeks, and one animal was added at 24 weeks, as the implanted iPSC-CP without a TEC detached from the defects in two of five implantation sites by 4 weeks after the initial operation.

The knees of five 34-week-old male nude rats were used to exhibit age-matched normal tissue for histological, radiological, and biomechanical analysis (sham) to compare to the treated animals with defects at 24 weeks postoperatively.

### Cell viability

Cell viability in fdTEC and basic TEC was assessed with the Live/Dead stain (Invitrogen, Carlsbad, CA, USA) and examined by epifluorescence microscopy.

### Histological analyses and evaluation of repaired tissue

The isolated tissues were fixed with 4% paraformaldehyde in phosphate-buffered saline (PBS) (pH 7.4) for a week, decalcified with EDTA, embedded in paraffin, and 3 µm sections were prepared from the center of the repair tissue. The sections were stained with hematoxylin and eosin (HE), Safranin O staining, and picrosirius red. Images were obtained using a DMi8 microscope (Leica, Germany) or a BX53/DP74 microscope (Olympus, Tokyo, Japan) equipped with a polarization filter.

Histological evaluation of tissue quality was performed using the modified O’Driscoll scoring system for cartilage and subchondral bone repair^19,41^. The sum of the cartilage and subchondral repair was also calculated from the scores.

### Immunohistochemistry

Paraffin sections of tissues from in vitro *and* in vivo experiments were prepared and subjected to immunohistochemistry. Cross-reactive monoclonal antibody to human vimentin (Anti-vimentin: ab16700, Abcom plc, UK, 1:200), type II collagen (Col II: Kyowa Pharma Chemical, Japan, 1:500), type X collagen (Col X: Abcom plc, UK, 1:100), IL1-β (IL1-beta Antibody; R12-2204, Assay biotechnology Company, CA, USA, 1:500), MMP-13 (Anti-MMP13; 18165-1-AP, Proteintech, Rosemont, IL, USA, 1:500), vascular endothelial growth factor (VEGF-A: ab1316, Abcom plc, UK, 1:200), rat/human CD31 (CD31: ab182981, Abcam plc, UK, 1:200), and human CD31 (CD31: ab76533, Abcom plc, UK, 1:200) were used as the primary antibodies. The de-paraffinized sections were incubated in 3% H_2_O_2_ for 10 min to block endogenous peroxidase activity, followed by incubation in proteinase K (DAKO, Glostrup, Denmark) for 5 min for antigen retrieval, and then blocking one histo (Nakarai, Japan) for 30 min to avoid non-specific binding of primary antibodies. Primary antibody was applied to each section and incubated for 1 h. Detection was then performed using Histofine® Simple Stain™ MAX PO (MULTI; Nichirei Bioscience, Tokyo, Japan) and Simple Stain™ DAB Solution (Nichirei Bioscience). All procedures were performed at room temperature, and during each step, the sections were washed three times with 0.1% Tween 20 in PBS for 5 min.

Human vimentin, IL-1β, MMP-13, and VEGF positive ratio were evaluated as follows: the square region of interest (ROI) with 100 µm long side was set at the distance of 0.75 mm from the surface and the bottom border of the defect on the center of the defect space (Fig. 8d), measured DAB stained positive and negative cells in the ROI (Fig. 8e) using Qupath software (https://qupath.github.io)^42,43^. The positive ratio was defined as (1).

$$^{\prime\prime} {\rm{Positive}}\; {\rm{ratio}}=\frac{{\rm{Positive}}\; {\rm{cell}}\; {\rm{number}}}{{\rm{Positive}}\; {\rm{cell}}\; {\rm{number}}+{\rm{negative}}\; {\rm{cell}}\; {\rm{number}}}\times 100 \% \,(1)^{\prime\prime}$$

### Radiological analysis using microfocus computed tomography (μCT)

The same five knees used for histological analysis were also used for radiological analysis (sham group: *n* = 5). All the specimens, which were obtained at 4, 12, and 24 weeks postoperatively, were scanned using the Skyscan μ-CT (Skyscan 1272, Bruker, Belgium). Scanning was performed using the following parameters: Camera binning = 2 × 2, Source Voltage (kV) = 70, Source Current (μA) = 142, Image Pixel Size (μm) = 10, Rotation Step (degree) = 0.4, Filter = Al 0.5 mm. Image analysis was performed by CTAN software (Version 1.18.8.0, Brucker). To evaluate the defect space, a rectangular interface region (2 × 3 × 1.5 mm) was defined as the Region of Interest (ROI; Fig. 8f, red rectangle). Bone volumes (BV), the area of new bone surface area (BS), the tissue volume (TV), trabecular number (Tb.N), trabecular thickness (Tb.Th), trabecular separation (Tb.Sp), structure model index (SMI) and degree of anisotropy (DA) inside the ROI were assessed.

### Biomechanical testing

Indentation testing was performed on the specimens using a self-made compression testing apparatus with an indentation probe consisted of a zirconia ceramic ball (Φ 1 mm). Each specimen was mounted on the sample stage of the compression tester and soaked in saline solution heated to 37 °C. Indentation testing was performed on the specimens at an indentation rate of 30 μm/s and indentation depth was 100 μm (Fig. 8g, h). Five knees from intact male athymic nude rats (34 weeks of age) were subjected to biomechanical testing and served as a normal control group (*n* = 5).

### Statistical analysis

Statistical analysis was performed using analysis of variance followed by post-hoc testing for the postoperative changes of total histological scores and biomechanical testing. The comparison of results for O’Driscoll score between each group was analyzed using the Kruskal–Wallis ANOVA test. The comparison of results for BV/TV, BS/TV, Tb.N, Tb.Th, Tb.Sp, SMI, DA, and biomechanical parameters between the groups were analyzed by ANOVA with the Bonferroni test. The results are presented as mean ± SD. The data were analyzed with BellCurve for Excel version 3.21 (Social Survey Research Information Co., Ltd., Tokyo, Japan) and significance was set at *p* < 0.05.

### Reporting summary

Further information on research design is available in the Nature Research Reporting Summary linked to this article.

### Supplementary information

Supplementary Figure 1

Reporting Summary Checklist

## Data Availability

The datasets generated during and/or analyzed during the current study are available from the corresponding author on reasonable request.

## References

[1] Huey DJ, Hu JC, Athanasiou KA (2012). Unlike bone, cartilage regeneration remains elusive. Science..

[2] Curl WW (1997). Cartilage injuries: a review of 31,516 knee arthroscopies. Arthroscopy..

[3] Peat G, McCarney R, Croft P (2001). Knee pain and osteoarthritis in older adults: a review of community burden and current use of primary health care. Ann. Rheum. Dis..

[4] Kon E (2011). Novel nano-composite multilayered biomaterial for osteochondral regeneration: a pilot clinical trial. Am. J. Sports Med..

[5] Gomoll AH (2010). The subchondral bone in articular cartilage repair: current problems in the surgical management. Knee Surg. Sports Traumatol. Arthrosc..

[6] Minas T, Gomoll AH, Rosenberger R, Royce RO, Bryant T (2009). Increased failure rate of autologous chondrocyte implantation after previous treatment with marrow stimulation techniques. Am. J. Sports Med..

[7] Wakitani S (1994). Mesenchymal cell-based repair of large, full-thickness defects of articular cartilage. J. Bone Joint Surg. Am..

[8] Nathan S (2003). Cell-based therapy in the repair of osteochondral defects: a novel use for adipose tissue. Tissue Eng..

[9] Shimomura K (2014). Osteochondral repair using a scaffold-free tissue-engineered construct derived from synovial mesenchymal stem cells and a hydroxyapatite-based artificial bone. Tissue Eng. Part A.

[10] Roelofs AJ, Rocke JP, De Bari C (2013). Cell-based approaches to joint surface repair: a research perspective. Osteoarthritis Cartilage.

[11] Takahashi K, Yamanaka S (2006). Induction of pluripotent stem cells from mouse embryonic and adult fibroblast cultures by defined factors. Cell..

[12] Takahashi K (2007). Induction of pluripotent stem cells from adult human fibroblasts by defined factors. Cell..

[13] Seki T, Yuasa S, Fukuda K (2011). Derivation of induced pluripotent stem cells from human peripheral circulating T cells. Curr. Protoc. Stem Cell Biol..

[14] Yamashita A (2015). Generation of scaffoldless hyaline cartilaginous tissue from human iPSCs. Stem Cell Rep..

[15] Ando W (2008). In vitro generation of a scaffold-free tissue-engineered construct (TEC) derived from human synovial mesenchymal stem cells: biological and mechanical properties and further chondrogenic potential. Tissue Eng. Part A.

[16] Ando W (2007). Cartilage repair using an in vitro generated scaffold-free tissue-engineered construct derived from porcine synovial mesenchymal stem cells. Biomaterials..

[17] Shimomura K (2010). The influence of skeletal maturity on allogenic synovial mesenchymal stem cell-based repair of cartilage in a large animal model. Biomaterials..

[18] Koizumi K (2016). Synovial mesenchymal stem cells from osteo- or rheumatoid arthritis joints exhibit good potential for cartilage repair using a scaffold-free tissue engineering approach. Osteoarthritis Cartilage.

[19] Shimomura K (2018). First-in-human pilot study of implantation of a scaffold-free tissue-engineered construct generated from autologous synovial mesenchymal stem cells for repair of knee chondral lesions. Am. J. Sports Med..

[20] Shimomura K (2021). Histological analysis of cartilage defects repaired with an autologous human stem cell construct 48 weeks postimplantation reveals structural details not detected by T2-Mapping MRI. Cartilage Cartilage..

[21] Scotti C (2013). Engineering of a functional bone organ through endochondral ossification. Proc. Natl Acad. Sci. USA.

[22] Farrell E (2011). In-vivo generation of bone via endochondral ossification by in-vitro chondrogenic priming of adult human and rat mesenchymal stem cells. BMC Musculoskelet Disord..

[23] Dennis SC, Berkland CJ, Bonewald LF, Detamore MS (2015). Endochondral ossification for enhancing bone regeneration: converging native extracellular matrix biomaterials and developmental engineering in vivo. Tissue Eng. Part B Rev..

[24] Jeske R, Bejoy J, Marzano M, Li Y (2020). Human pluripotent stem cell-derived extracellular vesicles: characteristics and applications. Tissue Eng. Part B Rev..

[25] Balbi C, Vassalli G (2020). Exosomes: beyond stem cells for cardiac protection and repair. Stem Cells.

[26] Herrmann, M. et al. Extracellular vesicles in musculoskeletal pathologies and regeneration. *Front Bioeng. Biotechnol*. in press (2021). 10.3389/fbioe.2020.624096. 10.3389/fbioe.2020.624096 PMC7855463 33553127

[27] Ghodrat S (2021). Stem cell-based therapies for cardiac diseases: the critical role of angiogenic exosomes. Biofactors.

[28] Germena G, Hinkel R (2021). iPSCs and exosomes: partners in crime fighting cardiovascular diseases. J. Pers. Med..

[29] Ashraf S, Walsh DA (2008). Angiogenesis in osteoarthritis. Curr. Opin. Rheumatol..

[30] Iwamoto M, Ohta Y, Larmour C, Enomoto-Iwamoto M (2013). Toward regeneration of articular cartilage. Birth Defects Res. C Embryo Today.

[31] García-Fernández L (2018). Osteochondral angiogenesis and promoted vascularization: new therapeutic target. Adv. Exp. Med. Biol..

[32] Yun HW, Choi BH, Park DY, Jin LH, Min BH (2020). Inhibitory effect of topical cartilage acellular matrix suspension treatment on neovascularization in a rabbit corneal model. Tissue Eng. Regen. Med..

[33] de Windt TS (2017). Allogeneic mesenchymal stem cells stimulate cartilage regeneration and are safe for single-stage cartilage repair in humans upon mixture with recycled autologous chondrons. Stem Cells..

[34] Craig W, David JW, Ming HZ (2003). A current review on the biology and treatment of the articular cartilage defects (part I & part II). J. Musculoskelet Res..

[35] Zevenbergen L (2018). Cartilage defect location and stiffness predispose the tibiofemoral joint to aberrant loading conditions during stance phase of gait. PLoS One..

[36] Ishikawa I, Sawada R, Yukio K, Tsuchiya T (2009). The effectivity of the novel serum-free medium STK2 for Proliferating human mesenchymal stem cells. Yakugaku Zasshi.

[37] Sawada R, Yamada T, Tsuchiya T, Matsuoka A, Microarray A (2010). analysis of serum-free medium on gene expression changes in human mesenchymal stem cells during the in vitro culture. Yakugaku Zasshi.

[38] Gottipamula S, Muttigi MS, Kolkundkar U, Seetharam RN (2013). Serum-free media for the production of human mesenchymal stromal cells: a review. Cell Prolif..

[39] International Society for Stem Cell Research. Successful Isolation and Expansion of Human Synovium-Derived Mesenchymal Stem Cells (MSCs) Grown Out from Tissue Explant by Using Chemically Defined Serum Free Media STK1® and STK2®. Updated June 2012. https://www.isscr.org/.

[40] Iimori Y, Morioka M, Koyamatsu S, Tsumaki N (2021). Implantation of human-induced pluripotent stem cell-derived cartilage in bone defects of mice. Tissue Eng. Part A.

[41] O’Driscol SW, Keele FW, Salter RB (1988). Durability of regenerated articular cartilage produced by free autogenous periosteal grafts in major full-thickness defects in joint surfaces under the influence of continuous passive motion. A follow-up report at one year. J. Bone Joint Surg. Am..

[42] Bankhead P (2017). QuPath: open source software for digital pathology image analysis. Sci. Rep..

[43] Humphries MP, Maxwell P, Salto-Tellez M (2021). QuPath: the global impact of an open source digital pathology system. Comput. Struct. Biotechnol..

