# Supplementary material for: Repair of osteochondral defects: efficacy of a tissue-engineered hybrid implant containing both human MSC and human iPSC-cartilaginous particles
Source: NPJ Regen Med. 2023 Oct 19;8:59. doi: 10.1038/s41536-023-00335-x (PMC10587071; doi:10.1038/s41536-023-00335-x)
Supplement: Supplementary file 1 — Supplementary Figure 1 [file 41536_2023_335_MOESM1_ESM.pdf]

Supplementary Figure 1.

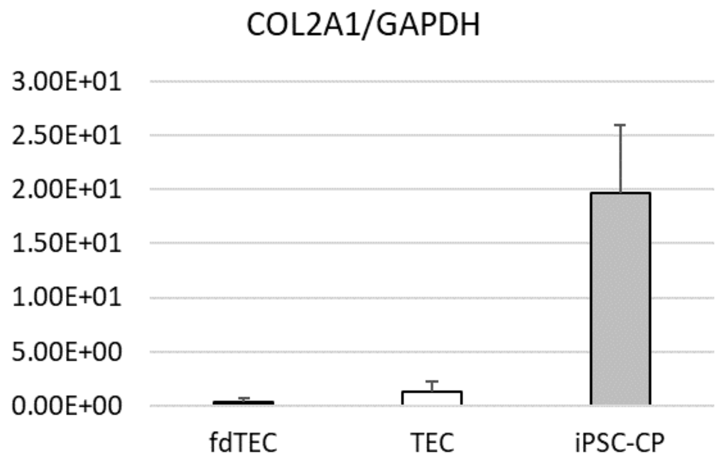

Supplementary Figure 1. Analysis of gene expression levels for collagen 2. Total RNA from the iPSC-CP, the TEC, and the fdTEC samples was extracted with an RNeasy Fibrous Tissue Mini Kit (Qiagen, Valencia, CA, USA) according to the manufacturer's protocol. Complementary DNAs (cDNA) were synthesized with SuperScript III (Invitrogen, Carlsbad, CA, USA) using random hexamers and 1.0 mg total RNA. Data represent mean ± SD.

In order to evaluate the comparative chondrogenic potential of the iPSC-CP, the TEC, and the fdTEC, the gene expression levels for the chondrogenic marker type II collagen, alpha 1 were assessed by qRT-PCR by TaqMan real-time polymerase chain reaction (qRT-PCR). Reactions were performed in 20 µl volumes containing 10 µl Taqman Fast Advanced Master Mix (Applied Biosystems, Carlsbad, CA, USA), 1 ul Taqman probes (COL2A1; Hs 00264051_m1), 1 ml cDNA template, and 8 ml nuclease-free water. A StepOnePlus Real-Time PCR instrument was used in the fast mode protocol (Applied Biosystems). The values for collagen II were normalized to glyceraldehyde-3-phosphate dehydrogenase (GAPDH; Hs02758891_g1).
