# Supplementary material for: Repair of osteochondral defects: efficacy of a tissue-engineered hybrid implant containing both human MSC and human iPSC-cartilaginous particles
Source: NPJ Regen Med. 2023 Oct 19;8:59. doi: 10.1038/s41536-023-00335-x (PMC10587071; doi:10.1038/s41536-023-00335-x)
Supplement: Supplementary file 2 — Reporting Summary Checklist [file 41536_2023_335_MOESM2_ESM.pdf]

nature portfolio

Corresponding author(s): Dr Waturo Ando

Last updated by author(s): Oct 3, 2023

Reporting Summary

Nature Portfolio wishes to improve the reproducibility of the work that we publish. This form provides structure for consistency and transparency in reporting. For further information on Nature Portfolio policies, see our Editorial Policies and the Editorial Policy Checklist.

Statistics

For all statistical analyses, confirm that the following items are present in the figure legend, table legend, main text, or Methods section.

n/a	Confirmed	
☐	☒	The exact sample size (*n*) for each experimental group/condition, given as a discrete number and unit of measurement
☐	☒	A statement on whether measurements were taken from distinct samples or whether the same sample was measured repeatedly
☐	☒	The statistical test(s) used AND whether they are one- or two-sided<br>*Only common tests should be described solely by name; describe more complex techniques in the Methods section.*
☐	☒	A description of all covariates tested
☐	☒	A description of any assumptions or corrections, such as tests of normality and adjustment for multiple comparisons
☐	☒	A full description of the statistical parameters including central tendency (e.g. means) or other basic estimates (e.g. regression coefficient) AND variation (e.g. standard deviation) or associated estimates of uncertainty (e.g. confidence intervals)
☒	☐	For null hypothesis testing, the test statistic (e.g. *F*, *t*, *r*) with confidence intervals, effect sizes, degrees of freedom and *P* value noted<br>*Give P values as exact values whenever suitable.*
☒	☐	For Bayesian analysis, information on the choice of priors and Markov chain Monte Carlo settings
☒	☐	For hierarchical and complex designs, identification of the appropriate level for tests and full reporting of outcomes
☒	☐	Estimates of effect sizes (e.g. Cohen's *d*, Pearson's *r*), indicating how they were calculated

Our web collection on statistics for biologists contains articles on many of the points above.

Software and code

Policy information about availability of computer code

Data collection	No software was used
Data analysis	No software was used

For manuscripts utilizing custom algorithms or software that are central to the research but not yet described in published literature, software must be made available to editors and reviewers. We strongly encourage code deposition in a community repository (e.g. GitHub). See the Nature Portfolio guidelines for submitting code & software for further information.

Data

Policy information about availability of data

All manuscripts must include a data availability statement. This statement should provide the following information, where applicable:

- Accession codes, unique identifiers, or web links for publicly available datasets
- A description of any restrictions on data availability
- For clinical datasets or third party data, please ensure that the statement adheres to our policy

The datasets generated and/or analyzed during the current study are available from the corresponding author on reasonable request.

Human research participants

Policy information about studies involving human research participants and Sex and Gender in Research.

Reporting on sex and gender	Sex was not considered in study design and this information has not been collected.
Population characteristics	NA
Recruitment	A patient were randomly recruited from patients received the arthroscopic surgery.
Ethics oversight	The organizations that approved on the study protocol was described in the manuscript.

Note that full information on the approval of the study protocol must also be provided in the manuscript.

Field-specific reporting

Please select the one below that is the best fit for your research. If you are not sure, read the appropriate sections before making your selection.

☒ Life sciences ☐ Behavioural & social sciences ☐ Ecological, evolutionary & environmental sciences

For a reference copy of the document with all sections, see nature.com/documents/nr-reporting-summary-flat.pdf

Life sciences study design

All studies must disclose on these points even when the disclosure is negative.

Sample size	We have previously reported the implantation of hydroxyapatite-TEC hybrid into the osteochondral defect in which the histological data of n=5 per group were presented with significant difference (Shimomura K, et al. Osteochondral repair using a scaffold-free tissue-engineered construct derived from synovial mesenchymal stem cells and a hydroxyapatite-based artificial bone. Tissue Eng Part A. 2014 Sep;20(17-18):2291-304.). The presented data in this study also demonstrated a significantly better total histological score using five samples per group. Therefore, we think this sample number is adequate.
Data exclusions	No data were excluded from the analysis.
Replication	All attempts at replication were sucessful
Randomization	Samples were randomly allocated into experiment groups.
Blinding	The investigators were blinded to group allocation during data collection.

Reporting for specific materials, systems and methods

We require information from authors about some types of materials, experimental systems and methods used in many studies. Here, indicate whether each material, system or method listed is relevant to your study. If you are not sure if a list item applies to your research, read the appropriate section before selecting a response.

Materials & experimental systems

n/a	Involved in the study	
☐	☒	Antibodies
☒	☐	Eukaryotic cell lines
☒	☐	Palaeontology and archaeology
☐	☒	Animals and other organisms
☒	☐	Clinical data
☒	☐	Dual use research of concern

Methods

n/a	Involved in the study	
☒	☐	ChIP-seq
☒	☐	Flow cytometry
☒	☐	MRI-based neuroimaging

Antibodies

Antibodies used	Provide supplier name, catalog number of all antibodies used in this study were described in this manuscript.
Validation	Each primary antibody for the species and application were validated.

Animals and other research organisms

Policy information about studies involving animals; ARRIVE guidelines recommended for reporting animal research, and Sex and Gender in Research

Laboratory animals	Ten-week-old male athymic nude rats (F344 NJcl-rnu/rnu; CLEA Japan, Fujinomiya, Japan)were used and described in the manuscipt.
Wild animals	This study did not involved wild animals.
Reporting on sex	Sex was not considered in study design and this information has not been collected.
Field-collected samples	The study did not involve samples collected from the field.
Ethics oversight	The organizations that approved on the study protocol was described in the manuscript.

Note that full information on the approval of the study protocol must also be provided in the manuscript.
